# Biochemical markers for prediction of the first half pregnancy losses: a review

**DOI:** 10.61622/rbgo/2024rbgo72

**Published:** 2024-09-06

**Authors:** Meruyet Kuspanova, Andrey Gaiday, Nurzhamal Dzhardemaliyeva, Maxat Tuganbayev, Maksym Gorobeiko, Andrii Dinets, Saule Bermagambetova, Zhanna Amirbekova, Gulshat Oraltayeva, Dinara Omertayeva, Akylbek Tussupkaliyev

**Affiliations:** 1 Department of obstetrics and gynecology, West Kazakhstan Marat Ospanov Medical University Aktobe Kazakhstan Department of obstetrics and gynecology, West Kazakhstan Marat Ospanov Medical University, Aktobe, Kazakhstan.; 2 Department of obstetrics and gynecology Asfendiyarov Kazakh National Medical University Almaty Kazakhstan Department of obstetrics and gynecology, Asfendiyarov Kazakh National Medical University, Almaty, Kazakhstan.; 3 Department of gynecology Atyrau Regional Perinatal Center Atyrau Kazakhstan Department of gynecology, Atyrau Regional Perinatal Center, Atyrau, Kazakhstan.; 4 Department of Surgery Institute of Biology and Medicine Taras Shevchenko National University of Kyiv Kyiv Ukraine Department of Surgery, Institute of Biology and Medicine, Taras Shevchenko National University of Kyiv, Kyiv, Ukraine.; 5 Department of Obstetrics, Gynecology and Perinatology Medical University of Karaganda Kazakhstan Department of Obstetrics, Gynecology and Perinatology, Medical University of Karaganda, Kazakhstan.; 6 Department of medical expertise Regional Perinatal Center Semey Kazakhstan Department of medical expertise, Regional Perinatal Center, Semey, Kazakhstan.

**Keywords:** Biochemical marker, Laboratory marker, Pregnancy, Prediction, Spontaneous abortion, Miscarriage, Missed abortion

## Abstract

**Objective:**

26% of all pregnancies end in miscarriage, and up to 10% of clinically diagnosed pregnancies, and recurrent pregnancy loss is 5% among couples of childbearing ages. Although there are several known causes of pregnancy loss in the first half, including recurrent pregnancy loss, including parental chromosomal abnormalities, uterine malformations, endocrinological disorders, and immunological abnormalities, about half of the cases of pregnancy loss in its first half remain unexplained.

**Methods:**

The review includes observational controlled studies (case-control or cohort, longitudinal studies, reviews, meta-analyses), which include the study of biochemical factors for predicting pregnancy losses in the first half, in singlet pregnancy. The Newcastle-Ottawa Scale (NOS) was used to assess the research quality.

**Results:**

Finally, 27 studies were included in the review, which has 134904 examined patients. The results of the review include estimates of β-human chorionic gonadotropin, progesterone, pregnancy-associated protein – A, angiogenic vascular factors, estradiol, α-fetoprotein, homocysteine and CA-125 as a predictors or markers of the first half pregnancy losses.

**Conclusion:**

It may be concluded that to date, research data indicate the unavailability of any reliable biochemical marker for predicting pregnancy losses in its first half and require either a combination of them or comparison with clinical evidence. A fairly new model shall be considered for the assessment of α-fetoprotein in vaginal blood, which may have great prospects in predicting spontaneous miscarriages.

## Introduction

The most common complication of the pregnancy is spontaneous abortion, at a term less than 20 weeks of gestation, which is frequently considered as the first half of pregnancy encompassing the first and part of the second trimesters.^1^According to published reports, a 26% of all pregnancies end in miscarriage, and up to 10% of clinically diagnosed pregnancies.^2^Recurrent pregnancy loss (RPL) is 5% among couples of childbearing age.^3^Moreover, 80% of miscarriages occur in the first trimester, with a reduced risk of miscarriage after 12 weeks of pregnancy.^4^It is generally assumed, that unprompted pregnancy losses occurring prior to the fetus are “physiological” phenomenon, preventing the conception of serious structural malformations or chromosomal aberrations incompatible with life from progressing to viability. Clinical studies confirm the availability of fetal malformations in 85% of cases and abnormal karyotype in 75% of cases, with spontaneous pregnancy losses in the first trimester.^5^Spontaneous pregnancy losses in the first half of pregnancy might be associated with such causes as molecular-genetic abnormalities in one of the parents,^6^immunological and immunogenic disorders,^7^thrombophilia^8^endocrine diseases,^9^fragmentation of sperm DNA,^10^endometrial embryo selectivity.^11^

It is worth to mention that approximately 50% of the cases of losses in the first half of pregnancy remain unexplained showing no connection to known causes such as recurrent pregnancy loss, including parental chromosomal abnormalities, uterine malformations, endocrinological disorders and immunological abnormalities.^12^

In addition to known factors, losses in the first half of pregnancy may also be related to the placenta’s disorders. In normal pregnancy, the earliest stages of development occur in the environment with a low oxygen concentration.^13,14^ A stable oxygen gradient between the decidua and fetoplacental tissue is also an important factor in trophoblast differentiation and migration, normal development of chorionic villi and angiogenesis.^15,16^Previous studies have shown that during normal pregnancy, physiological oxidative stress occurs in the placental tissue,^17^and changes in chorionic villi observed on the placenta periphery during the formation of fetal membranes are identical to those during miscarriage, indicating a common mechanism mediated by oxidative stress.^18^

Oxidative stress and increased oxygenation might alter the various placental proteins synthesis. Some studies indicate a relationship between in vivo oxygen concentration and inhibin-A and sFlt-1 concentrations in early pregnancy, which suggests that specific placental proteins may be regulated by intrauterine oxygen concentration. Fetoplacental angiogenesis begins on the 21st day after impregnation and continues during the first trimester, turning into a richly branched villous capillary bed.^19^Trophoblasts are regulated by specific angiogenic factors and also express such factors.^20^In particular, vascular endothelial growth factor (VEGF) and placental growth factor (PlGF) have been found to play an important role in stimulating neovascularization, while soluble fms-like tyrosine kinase (sFlt-1) acts as a growth inhibiting factor.^21-23^ A meta-analysis of 10 independent case-control studies showed that the polymorphisms rs1570360, rs3025039, rs2010963 and rs3025020 of VEGF were associated with the increased miscarriage risk.^24^ Abnormal placental vascularization with increased oxidative damage is a common cause of preeclampsia, fetal growth restriction due to placental insufficiency and early miscarriage.^13,25^Angiogenic factors in complicated early pregnancy were evaluated with some limitations, which requires further study of this issue and the identification of new prognostic models based on angiogenic factors expression.

The aim of this review study was to conduct a research literature analyses and to evaluate the possible role of angiogenic factors for predicting pregnancy losses in its first half of first trimester.

## Methods

The study was conducted within the framework of the scientific and technical project “Prediction of preeclampsia based on urinal PLGF and sFlt-1 concentrations: a multicenter cohort study” funded by the Ministry of Science and Education of the Republic of Kazakhstan, grant No. AP14869445.

The PRISMA statement and checklist was used to compile the study protocol and used for the study report.^26^The search for relevant studies was performed in the international databases Cochrane Central Register of Controlled Trials (CENTRAL), MEDLINE, EMBASE, Scopus, Web of Science, Google Scholar. The links of all known primary and review articles were examined to identify cited articles that were not detected by the electronic searching.

Medical subject headings (MeSH) were used to search in the title, keywords and abstracts for biochemical markers (“biochemical marker”, “biological marker”, “laboratory marker”, “marker”, “serum marker”) and also for pregnancy loss (“pregnancy”, “first trimester of pregnancy”, “prognosis”, “prediction”, “spontaneous abortion”, “miscarriage”, “missed abortion”) and were combined with the logical operators: AND and OR. Two authors independently searched databases without restrictions on the language and year of publication, looking through titles and abstracts. After that, repetitions in the found studies were removed by comparison, and the relevance of the selected studies was evaluated according to the studies complete text.

The study includes observational controlled studies (case-control or cohort, longitudinal studies, reviews, meta-analyses), which include the study of biochemical factors for predicting pregnancy losses in the first half, and in the singlet pregnancy. Reviews are included in the original search to check for supporting links. The assessment of the biochemical factors level is accepted both before the onset of clinical evidence of pregnancy loss and with clinical symptoms. Outcome (*i.e.* pregnancy loss in the first half) was evaluated according to the recommendations of the European Society of Human Reproduction and Embryology (ESHRE), after laboratory, clinical and ultrasound diagnostic methods (U/S) of uterine pregnancy: uterine bleeding in the first half of pregnancy accompanied by complete or incomplete expulsion of the gestational sac/fetus from the uterine cavity confirmed by ultrasound; deceased gestational sac/fetus without clinical evidence, confirmed by ultrasonic examination. With abundant uterine bleeding requiring instrumental management, positive serum chorionic gonadotropin, without ultrasound confirmation of the presence of a gestational sac/fetus in the uterine cavity, with the exception of ectopic pregnancy.

Comments, editorials, correspondence with the editorial board, case series (as determined by the study’s authors), reports and animal studies, outcomes not consistent with the ESHRE criteria were excluded from the study.

The Newcastle-Ottawa Scale (NOS) was used to assess the research quality.^27^The articles have been classified as of high quality (≥ 5 points) or of low quality (<5 points). The study includes all articles that scored ≥ 5 points. Two authors independently reviewed articles according to the NOS scale. In case of disagreement between the authors on the study assessment, the third author conducted an independent assessment according to the NOS scale, as a result of which a decision was made to assess the study quality and its inclusion in the study.

Extraction of the study outcome indices, which are indicated in the selected articles, were carried out according to the following criteria: year of publication, first author, study design, study population, number of subjects in the pregnancy loss group and control group, trimester or gestation age.

## Results

**Figure 1:**
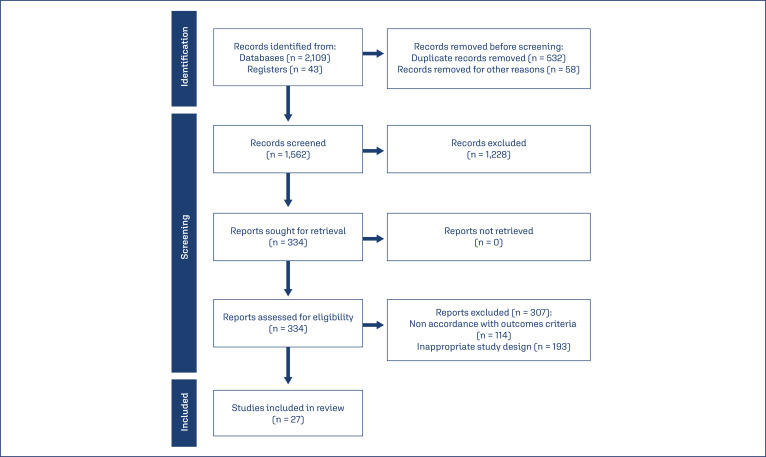
Flow chart illustrating the selection process of the studies for the review

According to the search a 2,152 research papers were identified. Analyses of the abstracts and full-text articles revealed 27 studies which were included in the review. These 27 studies gather 134904 examined patients as showed in figure 1.

### β-hCG (β-human chorionic gonadotropin)

According to Stewart et al.,^28^ the β-hCG subunit may be detected in the plasma of a pregnant woman as early as eight days after ovulation. Quantitative levels of β-hCG may provide useful information about early pregnancy. The results of research by Barnhart et al.^29^ indicated that in women with a viable intrauterine pregnancy, the initial levels of β-hCG should be within 48 hours by at least 49% in case of β-hCG of 1500 mMU/ml, by 40% in case of β-hCG from 1500 to 3000 mMU/ml, and by 33% in case of β-hCG exceeding 3000 mMU/ml. Lower concentrations of β-hCG increase to suggest early pregnancy abortion or ectopic pregnancy. Meta-analysis by Pillai et al.^30^ showed role of low β-hCG for prognosis of pregnancy abortion with β-hCG sensitivity of 44% (95% CI 17-75%), specificity of 86% (95% CI 80-91%), positive likelihood ratio of 3.37 (95% CI 1.98–5.74%) and negative likelihood ratio of 0.63 (95% CI 0.36–1.11). The level of hCG in blood increases rapidly with a maximum level of 50 000–1 00 000 IU/ml attained at about 8–10 weeks of gestation. The consistent nature of this pattern has made quantitative determinations of hCG a valuable tool in the clinical assessment of early pregnancy abnormalities.^30^ By approximately 10 weeks of pregnancy, the level of β-hCG usually stabilizes or decreases and cannot be used as marker for pregnancy, therefore serial ultrasound is the preferred diagnostic tool at that period of pregnancy.^31^

### Progesterone

Measurement of progesterone in serum is useful for distinguishing between early viable or non-viable pregnancy, especially in conditions of inconclusive ultrasonic examination. In a meta-analysis by Verhaegen et al.,^32^ evaluating the accuracy of a single progesterone test to predict the pregnancy outcome in women with bleeding in the first trimester, showed that its concentration was less than 6 ng/ml (19.1 nmol/l) reliably excludes viable pregnancy with a negative predictive value of 99%. Low progesterone concentrations do not allow distinguishing intrauterine pregnancy from extra-uterine pregnancy.^32^In the meta–analysis, Pillai et al.,^30^ devoted to the study of serum progesterone for predicting fetus wastage, showed the sensitivity of 30% (95% CI 2-87%), specificity of 86% (95% CI 78-91%), the positive likelihood ratio of 2.24 (95% CI 0.32-15.80%) and the negative likelihood ratio of 0.81 (95% CI 0.35–1.86).

### PAPP-A (Pregnancy-associated protein - A)

Since PAPP-A is regularly measured in all women and is relevant to pregnancy outcomes; expression of this protein is performed by cost-effective method and therefore might be used to predict the pregnancy losses. Two recent studies have examined the relationship between low RARP-A levels and subsequent spontaneous pregnancy losses.^33,34^In 2012, Hanita et al.^34^ performed a prospective cohort study on 42 women at risk of miscarriage and 40 women were in the control observation group. Out of 82 women, on 9 (11%) women the authors found that the average PAPP-A level was significantly lower in the group with pregnancy losses, compared with the observation group (0.78 multiple of median (MoM) vs. 1.00 MoM *P* < 0.05).^34^In a 2011 a cohort study was performed by van Ravenswaaij et al.^33^ among 28,566 women, showing a low level of RARP-A below the 5th percentile as predictor of subsequent miscarriage (odds ratio 14.53; 95% CI from 10.44 to 20.22). These recent studies suggest that low levels of PAPP-A may be associated with subsequent pregnancy loss before the fetus becomes viable. A meta-analysis by Pillai et al.^30^ has showed that PAPP-A had low and wide sensitivity in the range from 25 to 64%, but high specificity in the range from 88% to 94%. As a result of another meta-analysis by Hadizadeh-Talasaz et al.,^35^ it was concluded that PAPP-A might not be recommended for routine prediction of fetal loss and further studies with a combination of other biomarkers are needed. Further research results showed the prognostic significance of PAPP-A for pregnancy loss sensitivity 57% (95% CI 53-63%), specificity 83% (95% CI 80-85%), positive the likelihood ratio is 3.52 (95% CI 2.44-5.07), the negative likelihood ratio is 0.54 (95% CI 0.37–0.79) and the diagnostic odds ratio is 6.95 (95% CI 3.58–13.50).^35^ Dugoff et al.^36^ in a trial study FaSTER has showed that although a low level of PAPP-A is associated with unfavorable pregnancy outcomes; it is a poor predictor of such outcomes.

### Angiogenic vascular factors (VEGF – endothelial growth factor, PLGF – placental growth factor, sFlt-1 – soluble tyrosine kinase - 1)

Recent data have demonstrated that altered expression of VEGF family members may be a contributing factor to pregnancy loss and recurrent pregnancy loss.^37,38^The results of the study of the expression of VEGF and its receptors in women with unexplained recurrent pregnancy loss have presented that during the average-late secretory phase and in the peri-implantation period, decreasing of the expression of VEGF-A in vascular smooth muscle cells, endothelial cells and glandular epithelial cells decreased, while the expression of VEGFR-1 in stromal cells was also decreased.^39,40^On the other hand, patients with recurrent pregnancy loss had higher expression of VEGF in the lumen epithelium, glandular epithelium and endometrial stroma during embryo implantation as showed by Chen et al.^41^Pang et al.^37^ has also found that serum levels of VEGF and sFlt-1 in women with pregnancy losses were significantly higher as compared to women with normal pregnancy.^37^In a study by Muttukrishna et al.^42^ it was found that the average level of serum sFlt-1 was significantly decrease by 86% in the group patients with miscarriage as compared to the healthy patients in control group. Serum PLGF levels were lower in patients with miscarriage as compared to patients who had full-term live-born child.^42^In study by Kaitu’u-Lino et al.,^4^ a significant decrease (35%) of circulating sFlt-1 in serum was found in the miscarriage cohort as compared to women who gave birth to a live child at term 1.0 (0-3.3) MoM vs. 0.65 (0-1.94) MoM. In that study no changes were observed in the circulating serum PlGF between the groups of pregnant women with miscarriage and with the normal gestation course. It is also worth to mention that Kaitu’u-Lino et al.^4^ also did ROC analysis, showing the area under the curve (AUC) for sFlt-1 was 0.66 (p = 0.03) associating with pregnancy loss. Consequently, despite the statistically significant difference in sFlt-1 between the miscarriage cohort and the control group, sFlt-1 has low sensitivity and specificity for predicting miscarriage.^4^In a study by Jayasena et al.^43^ dedicated to the investigation of circulating angiogenic factors associated with late miscarriage, it was found that the levels of sFlt-1 and PLGF were lower in women who later suffered a miscarriage compared with uncomplicated pregnancy. Logistic regression modeling showed that increased sFlt-1 (odds ratio 0.15, 95% CI 0.08-0.26, p = 0.0001) and PLGF (odds ratio 0.02 95 CI 0.01-0.05, p = 0.0001) were associated with the lower risk of miscarriage after adjusting for age, BMI, gestational age, smoking and blood pressure. The combination of sFlt-1 and PLGF did not increase the prediction accuracy.^43^ In another study, by Daponte et al.,^44^ it was found that the concentration of serum PLGF and sFlt-1 was lower both in ectopic pregnancy (14.6±3.42/178.16±76.03 pg/ml) and in pregnancy losses (16.25±4.73/399.42±337.54 pg/ml) compared with uncomplicated pregnancy (21.64±5.68/1390.32±655.37 pg/ml). In women with viable uterine pregnancies, the expression of PLGF and Flt-1 genes were significantly lower in women with miscarriages and extrauterine gestation.^44^In another study by Stohl et al.^45^ it was found that the average level of sFlt-1 in serum was higher in the first trimester of pregnancy than in non-pregnant controls, and high levels of sFlt-1 in the first trimester were associated with spontaneous miscarriages AUC 0.755 (95% CI 0.606-0.904) with the cutoff level of 0.1865 ng/ml, sensitivity 76.9%, specificity 73.7%, positive likelihood ratio 0.243, negative likelihood ratio 0.966, odds ratio 9.35 (95 CI 2.41-36.22).

### Estradiol

In a systematic review with meta-analysis by Pillai et al.,^30^ four studies involving 244 women were included, in which estradiol in serum was studied to predict the outcome in women at miscarriage risk (30). Data analysis showed estradiol as a good prognostic marker for pregnancy loss with sensitivity of 45% (95% CI 6-90%), specificity of 87% (95% CI 81-92%), positive likelihood ratio of 3.72 (95% CI 1.01–13.71) and negative likelihood ratio of 0.62 (95% CI 0.20–1.84). Deng et al.^46^ showed that at 5-6 weeks of gestation cut-off level for estradiol associated with a miscarriage was 320 pg/ml with an AUC under the ROC curve equal to 0.709 (95% CI 0.793- 0.938, *p* = 0.000), a diagnostic threshold value at 7-9 weeks of gestation of 576 pg/ml, sensitivity 0.804, specificity 0.829. As a result of the conducted studies, it was revealed that low estradiol levels in the terms of 7-9 weeks of pregnancy may be used to predict miscarriage in the first trimester.^46^

### α-fetoprotein

α-fetoprotein (AFP) is a product of specific fetal tissues and neoplastic cells of hepatocytic or germ origin in adults.^47^In a retrospective study by Hu et al.,^48^ it was determined that elevated AFP in maternal serum (≥ 2.5 times MoM) increases the risk of miscarriage (OR 20.81), with the frequency of 7.46%. The study by Mor et al.^49^ devoted to the identification of miscarriage by AFP concentration in vaginal blood showed mean concentrations of AFP in vaginal blood of 192.2 ng/ml (range 9.2-195 ng/ml) and in maternal serum 6.2 ng/ml (range 0.9-211 ng/ml) for miscarriages/incomplete miscarriages, whereas they were only 73.1 (range 1.5-280 ng/ml) and 25.2 ng/ml (range 18.7-73.3 ng/ml) for groups of high risk of pregnancy loss and cerclage, respectively.

### Homocysteine

Homocystein (Hcy) has an impact on the risk for the pregnancy loss. According to recent research by Dai et al.^50^ hyperhomocytoseinemia (HHcy) was showed to be associated with various pregnancy complications, including recurrent pregnancy loss (RPL).^50,51^According to published data, one third of the patients with RPL were diagnosed with Hhcy.^52^HHcy may cause damage to endothelial cells, and therefore it may also cause the damage the blood vessels of the placenta, leading to pregnancy losses. Nelen et al.^53^ has found that an increase in fasting Hcy (≥18.3 mmol/L) and Hcy after exercise (≥61.5 mmol/L) were associated with RPL. Methylenetetrahydrofolate reductase (MTHFR) gene mutation is related to elevated total homocysteine (tHct) expressions, in particular, among women with low folate intake.^50^Obviously, compared to women with the MTHFR 677C>C or 677C>T genotypes, women with the MTHFR 677T>T genotype showed higher levels of Hcy.^54^However, a study by Creus et al.^55^ has demonstrated that there were no significant variances in the expression of homozygous and heterozygous mutations of the MTHFR gene between 60 unexplained RPL and 30 normal pregnant women. Similar findings were also reported by Zammiti et al.^56^ in 350 women with RPL and 200 healthy showing any significant correlation between Hcy and RPL. Zammiti et al.^56^ also concluded that mutations of maternal genes lead to renal or cardiac embolic diseases; however, these genetic abmormalities may not have the same harmful effect on placental circulation during the pregnancy first trimester.

### CA-125

CA-125 is an antigenic determinant of a glycoprotein with a high molecular weight and is produced by normal and malignant cells of various origins (predominant in tissues originating from the Mullerian epithelium). In a study by Barooti et al.,^57^ it was found that the level of CA-125 in miscarriage was 27.70 ± 7.50 IU/mL, as compared with controls 22.51 ± 6.82 IU/mL (P<0.001). In other study, Fiegler et al.^58^ indicates that the level of CA-125 ≥ 43.1 U/ml might be associated with an increased risk of miscarriage, and in the presence of vaginal bleeding during pregnancy ≥ 3 days CA-125 of 66.5 U/ml might be considered as a cut-off value for the risk determination of the pregnancy loss. The meta-analysis by Pillai et al.^30^ showed that CA-125 for miscarriage prediction was associated with a sensitivity of 90% (95% CI 83-94%), specificity of 88% (95% CI 79-93%), the positive likelihood ratio of 7.85 (95% CI 4.23–14.60) and the negative likelihood ratio of 0.10 (95% CI 0.05–0.20).

## Conclusion

Analyses of the published series to indicate the unavailability of any distinct biomarker for predicting pregnancy losses in its first half, which is required either a combination of them, for example, estradiol with progesterone or β-hCG with progesterone, or comparison with clinical evidence, for example vaginal bleeding. A fairly new model shall be considered the assessment of α-fetoprotein in vaginal blood, which may have great prospects in predicting spontaneous miscarriages. In order to reliably interpret biochemical markers in the pregnancy early stages, normograms for a specific gestational age are required, and predefined thresholds shall be important for the further research design.
